# The effects of age and gender and elite levels on perceptual–cognitive skills of adolescent badminton athletes

**DOI:** 10.3389/fpsyg.2024.1415693

**Published:** 2024-07-02

**Authors:** Kuo-Cheng Wu, Yu-Lung Lee, Shiau-Cheng Chen

**Affiliations:** ^1^Graduate Institute of Sports Training, University of Taipei, Taipei, Taiwan; ^2^Faculty of Medicine, Semmelweis University, Budapest, Hungary

**Keywords:** information processing, reaction time, working-memory, adolescent, perceptual-cognitive

## Abstract

**Introduction:**

This study aimed to examine perceptual-cognitive skills across age, gender and elite levels of badminton adolescent athletes.

**Methods:**

A total of 57 badminton athletes divided into junior high school athletes (age = 13.36 ± 1.14 years, females = 22, males = 11) and senior high school athletes (age = 16.25 ± 0.84 years, females = 11, males = 13) were evaluated using a cognitive component skills approach. Elite levels were classified as semi-elite (*n* = 29, score = 3.23) and competitive elite (*n* = 28, score = 5.84) levels. Each group completed a cognitive test, including an evaluation of their capacity for Corsi block-tapping (CCT) and spatial priming tasks (SPT).

**Results:**

No gender effects were found in the perceptual skills of the adolescent players, and the age effect was consistent across gender. For the elite levels, the perceptual-cognitive skills of SPT of reaction time was performed equally in the groups of semi-elite and competitive players, however, in the CCT Span of working-memory (WM), competitive-elite players outperformed semi-elite players.

**Conclusion:**

We found that perceptual-cognitive skills of WM play crucial roles in the open-skill sports of badminton. Thus, when developing advanced skills to higher elite levels in adolescent players, perceptual-cognitive skills should be considered.

## 1 Introduction

Elite athletes typically exhibit superior perceptual and cognitive skills compared to semi-elite athletes or nonathletes owing to the physical training and cognitive stimulation provided by sports (Alves et al., 2013). Elite athletes gain a competitive edge by developing cognitive skills and strategies through deliberate practice, enhancing their ability to process information more efficiently (Eccles, 2006; Ericsson, 2006; Furley et al., 2015). Studies conducted over time have shown that sports experts have greater perceptual-cognitive skills in picking up information (Mann et al., 2007). Furthermore, types of sports in terms of open or closed skills (Schmidt et al., 2018) may affect neurocognition, which is a crucial element for efficient elite levels (Eccles, 2006; Ericsson, 2006; Furley P. and Memmert, 2010), specifically in open-skill sports, the distinctive neurocognitive changes are induced by sports types (Mann et al., 2007; Tsai et al., 2017; Hung et al., 2018). Badminton, classified as an open-skill sport (Goodway et al., 2021), can be mastered at the elite level by refining various stroke strategies. This sport demands rapid judgment, which in turn fine-tunes the tactical decision-making process of the human brain, thereby optimizing performance. This allows confident decisions to be made within seconds (Shaw, 1989). In perceptual and cognitive studies relative to badminton, Jin et al. (2011) suggested that badminton players have higher C1 wave amplitudes, reflecting their correct decision-making and anticipatory abilities. Wang et al. (2015) argued that badminton players perform better in basic visuospatial cognitive operations than nonathletes. Furthermore, Hung et al. (2018) studied acute running and badminton exercises and found that badminton exercise has a relative impact on Brain-Derived Neurotrophic Factor (BDNF) and executive function.

Similar to other sports, badminton success requires both physical and perceptual abilities. Advanced perceptual skills must be developed to ensure that the execution of skills is both effective and proficient, one needs to have advanced perceptual skills (Mori et al., 2002). Recent studies and theoretical developments in the fields of cognitive science and neuroscience have identified positive correlations between complex cognitive functions and attention, selective attention, and working-memory (WM) (Kane et al., 2001; Kane and Engle, 2003). These functions are recognized as interrelated fundamental cognitive processes (Ku, 2018) and share similar underlying neural mechanisms. In sports, the effectiveness of attentional resource transitions often plays a critical role in competitive sports, and elite athletes appear to be able to operate efficiently in the transition of information processing (Eccles, 2006; Ericsson, 2006; Furley P. and Memmert, 2010), allowing motor skills to be performed appropriately. A meta-analysis of studies of athletes of different skill levels revealed that athletes with higher skill levels performed better cognitive skills than athletes with lower skill levels (Tomporowski and Pesce, 2019), however, the elements of cognitive skills in the higher skills athletes have not yet clear (Kalén et al., 2021).

The WM model not only facilitates information storage but also integrates cognitive control and attention mechanisms (Baddeley, 1992; Baddeley, 2003), allowing the model to incorporate complex behaviors and their underlying processes. Athletes’ performance is influenced by their WM capacity when engaging in two attention-demanding tasks when executing a motor action while processing external stimuli. Other studies have demonstrated a reciprocal relationship between attention and WM (Furley P. A. and Memmert, 2010; Furley and Wood, 2016).

Attention encompasses all cognitive processes and regulates the activation of internal and external representations (Posner and Petersen, 1990; Knudsen, 2007). This regulation could allow the outsources of stimuli to enter the WM (Atkinson and Shiffrin, 1968), and WM influences attentional control (Soto and Humphreys, 2007). In this way, higher WM capacity can be preserved as active memory. Therefore, WM plays a crucial role in the comprehension of human cognition in real-life contexts. Empirical studies on athletes support the role of WM in decision-making, which relies on controlled attention (Furley and Memmert, 2012). Furley and Memmert (2013) provided evidence that WM plays a crucial role in directing and allocating attention in athletes. In addition, the WM capacity indicates an individual’s ability to control attention across domains (Kane et al., 2007).

WM is a cognitive system comprising multiple elements. This system incorporates the executive in the center, along with two subsidiary mechanisms: the phonological loop and the visuospatial sketchpad (Baddeley and Hitch, 1974; Baddeley, 1992; Baddeley, 2003; Baddeley, 2007). They argued that WM not only accurately predicts a person’s ability to control their attention but also those who may falter in high stress during the games (Furley and Memmert, 2012). Conversely, those with low WM struggled to adapt their strategies to meet the game’s demands, and those who scored high on WM evaluations displayed a heightened ability to concentrate. Supported by empirical studies on athletes, WM capacity plays a role in decision-making in tactical games, which depends on controlled attention (Furley and Memmert, 2012). Some researchers have argued that sports experts do not achieve higher WM scores (Furley and Wood, 2016; Buszard and Masters, 2018). However, researchers have not differentiated their findings across expertise (Swann et al., 2015).

In the context of performance, the computer-based findings of Furley and Memmert (2012) suggest that WM can predict the ability to resolve response competitions. Athletes’ performance prediction may be related to their better selective attention (Abernethy, 1988; Schumacher et al., 2018), focusing on crucial information and performing cognitive processing in a complex environment (Johnston and Dark, 1986). However, some studies have demonstrated that basketball players perform the same cognitive skills as nonathletes (Furley P. and Memmert, 2010); further research should clarify these mechanisms.

Regarding the maturation of athletes’ age of development, studies indicated that age affects the reaction time (RT) in the different age groups. According to Petrakis (1985), 10-year-old players achieved faster detection time compared to 8-year-olds. Benguigui and Ripoll (1998) stated that age affects the timing of the initiation of the response, and RT decreases as age increases into adulthood. Williams et al. (2002) examined youth tennis players at different age levels and showed that the younger age groups performed slower response initiation times than the older groups. On the other hand, when consider gender effect in RT, research indicates that between the ages of 4 and 9, hormonal differences lead to boys having faster detection times than girls (Petrakis, 1985). This advantage continues into adulthood, with males consistently outperforming females in detection tasks (Brady, 1996; Caccese et al., 2020). As mentioned above, both age and gender significantly influence RT.

From the perspective of working memory, considering the effects of gender and age, Tremblay et al. (2022) found significant differences among youth athletes in terms of body measurement, elite levels, and perceptual and cognitive skills. Ward and Williams (2003) found that in terms of cognitive skills related to working memory, age was a more reliable predictor of structured memory recall than motor ability. Similarly, Farrell Pagulayan et al. (2006) identified significant differences based on grade, reinforcing the influence of age on memory performance. Neuroscientific research indicates gender differences in WM, which are seen in the activation patterns: females predominantly activate the limbic and prefrontal areas in contrast to males, who demonstrate a more dispersed activation pattern, notably in the parietal areas (Hill et al., 2014). Considering gender in growth and development, the rapid growth stage of height in females is at 11 years of age, and after 16 years of age, height development enters the end stage, it can be seen that the growth and maturation of female in adolescent, female athletes is faster than that of male athletes in the adolescent stage (Goodway et al., 2021). Nevertheless, the research of WM in adolescents found no significant gender differences (Farrell Pagulayan et al., 2006; Rigoli et al., 2012); Research by Rigoli et al. (2012) examining motor coordination, academic achievement, and working memory (WM) found no gender differences among adolescent participants. Similarly, Farrell Pagulayan et al. (2006) reported no significant gender effects in their study. Moreover, studies have indicated that adult males respond more quickly to WM tasks, with no observed differences in accuracy between the gender (Loring-Meier and Halpern, 1999).

As previously stated, studies investigating RT and WM in badminton athletes have revealed that various factors, including age, gender, influence the perceptual-cognitive skills (Brady, 1996; Loring-Meier and Halpern, 1999; Farrell Pagulayan et al., 2006; Caccese et al., 2020). Furthermore, only a few articles have assessed cognitive skills in childhood and adolescence to identify their role in the field of sports (Ward and Williams, 2003; Ishihara et al., 2019; Murr et al., 2021) and future sporting success (Kalén et al., 2021). Further studies should incorporate controls for age-difference groups with high fitness levels to enable meaningful comparisons (Hillman et al., 2008, 2015).

To the best of our knowledge, no previous research has investigated the perceptual-cognitive skills of experienced badminton players with regard to gender, age and elite levels among student-athletes. Therefore, this study aimed to examine the effects of age and gender in adolescent badminton players on perceptual and cognitive skills in RT and WM. Additionally, we explored whether elite levels could be influenced by perceptual and cognitive skills.

Based on our findings, we hypothesized that age and gender impact perceptual-cognitive skills in adolescent, and that badminton athletes with greater perceptual and cognitive skills exhibit better elite levels.

## 2 Materials and methods

### 2.1 Participants

A total of 57 adolescent badminton athletes were recruited. The participants were divided into two main groups based on academic levels, reflecting their developmental stages (males rapid growth in 16–18 years with senior high stage, females rapid growth in 13–16 years with junior high stage), with consideration for gender-specific growth patterns. In Taiwan, this approach extends to badminton competition and promotes fairness by acknowledging these differences. The junior high school group consisted of adolescent athletes who averaged 13.36 ± 1.14 years in age, with 22 females and 11 males, while senior high school group averaging 16.5 ± 0.84 years in age comprised 11 females and 13 males. This study was conducted in accordance with the Declaration of Helsinki and approved by the Institutional Review Board of the University of Taipei (protocol code: UT-IRB No. IRB-2020-073, February 9, 2021–2024) and received written informed consent from the participants’ legal guardians.

### 2.2 Procedures

All participants were tested in a laboratory and the experiment lasted for approximately 40 min. Prior to the test session, the participants were required to complete a questionnaire. Badminton players answer these questions to obtain scores, which are then used the formula by Swann et al. (2015) to calculate their competitive scores. All participants responded on a 1,428.4 × 803.5 mm and 8 ms display to acquire reaction times. The testing methods for spatial priming and WM capacity are described as the cognitive component test section.

#### 2.2.1 Classify the athlete’s elite levels

In this questionnaire, they were asked to rate their sport performance based on their career competition results and using the classification developed by Swann et al. (2015) for adolescent players, in which competitive scores were calculated as per the existing literature. The competitive scores of adolescent athletes are judged based on researcher questions developed by Swann et al. (2015), which consist of two parts: competition within the athlete’s country and competition within the sport globally. In a country, an elite athlete’s status is determined by the level of competition within their sport, depending on factors such as the country’s size and the sport’s popularity. Similarly, global competition in the sport determines an athlete’s status, considering the number of competitors worldwide and the effort required to excel. The resulting scores were subsequently classified as follows: 1–4 = Semi-Elite athletes, 4–8 = Competitive Elite athletes, 8–12 = Successful Elite athletes, and 12–16 = World-Class Elite athletes. Table 1 contains the elite levels and scores of the participants. The higher level of the elite, the higher the score the athlete received.

**Table 1:** Elite levels scores of age and gender badminton players.

Age/academic phase	Gender	*N*	Age	Training years	Elite athlete’s levels (Swann et al., 2015)				
					Semi elite	Competitive elite	Successful elite	World class elite	Scores
Junior	Female players	22	13.64 ± 1.13	6.29	15	7	1	0	3.67
	Male players	11	12.82 ± 0.98	4.27	8	3	0	0	4.32
Senior	Female players	11	16.73 ± 0.90	8.77	1	10	5	0	5.75
	Male players	13	15.85 ± 0.55	8.84	5	8	1	0	5.05

Elite athlete’s levels score, semi elite 1–4, competitive elite 4–8, successful elite 8–12, world class elite 12–16.

#### 2.2.2 Cognitive component test

##### 2.2.2.1 Spatial priming task: reaction time

SPT evaluates rapid attention and response switching in a changing signal. The experimental procedure was a SPT from a The Psychology Experiment Building Language (PEBL) test battery (Mueller and Piper, 2014). Correlations have been observed between RT and perceptual speed (Carlson et al., 1983); thus, we used a SPT of RT to test athletes’ attention ability. In this program, the participants were presented with a 3 × 3 array of squares on a screen. Whenever the program was executed, it ran a total of 54 trials, according to Nelson (2013) PEBL technical report 2013-1, there are significant difference between five conditions. During each trial, the participants were instructed to respond with both speed and precision when one of the nine squares turned blue (target square). Before the target square appeared, one of the squares briefly flashed yellow (visual cue). The cue was not always in the same location as the target square, and the SPT measurement variables were as follows: in some trials, there was no cue (NC), the cue appeared in the same row (SR), the cue appeared in the same column (SC), the cue appeared in the same square (SS), and the cue appeared in a different row and column (OS). A total of 54 trials were conducted each time the program was conducted. Thus, we can compare the cueing conditions with research conducted using this task in other subjects, specifically in different sports.

##### 2.2.2.2 Working-memory: capacity of Corsi block-tapping

Widespread usage in cognitive psychology is found in the operation, counting, and reading span tasks (Conway et al., 2005). Counting span tasks to assess working memory. These tasks typically require multiple subcomponents of executive functions (Diamond, 2013). In the present study, the Corsi block-tapping task (CCT) served as a measure of spatial ability. This task demonstrates the sensitivity of the dependent measure, which has been previously employed to assess developmental differences among children and adolescents (Farrell Pagulayan et al., 2006), Additionally, it has been applied in the field of sports (Furley P. and Memmert, 2010). To assess WM capacity, we used the Spatial Working Memory (SWM) task, a computerized version of the CCT. The participants reproduced the color-changing sequences by touching the corresponding boxes on the screen. The task began with a two-box sequence and advanced to nine boxes. The outcome measure is the span length, which indicates WM capacity. The (CCT) includes variables such as Block Span (BS), Total Score (TS), Total Correct Trials (TC), and Memory Span (MS).

### 2.3 Statistical analysis

We used a two-way MANOVA for measures, with gender and age as independent variables and within the SPT task as dependent variables. Wilks’ lambda values for group differences in a set of dependent variables were determined (Tabachnick et al., 2013). Similarly, a two-way MANOVA was used for the task of the CCT, and we examined the main effects and interactions of both gender and age. Effect sizes were expressed as partial eta-squared values in MANOVA (small ≥0.01, medium ≥0.06, large ≥0.14), with Cohen’s d (small ≥0.2, medium ≥0.5, large ≥0.8) indicating mean group differences. Significance was set at 0.05 (Cohen, 2013). The groups of elite levels were divided into semi-elite and competitive-elite categories, with age serving as a significant factor. Therefore, the MANCOVAs were employed to analyze the association between WM and the same statistical method was used for spatial priming tasks. Based on the classification devised by Swann et al. (2015), the elite levels of youth athletes was divided into two groups: semi-elite and competitive-elite. SPSS Statistics software (version 26) was used to analyze the data, and the significance level was set at *p* < 0.05.

## 3 Result

The normality of the distribution of continuous variables was assessed using the Kolmogorov–Smirnov (K–S) Normality Test, which revealed that the SPT outcomes did not significantly deviate from a normal distribution (*p* > 0.05). In the CCT, the normality of the distribution was rejected (*p* < 0.05). so a generalized rank-order method for nonparametric two-way MANOVA testing of CCT data was used (Thomas et al., 1999). Reliability of within-subject variation in SPT typical error is 58.32 (10%); CCT typical error is 1.024 (14%) (Hopkins, 2000).

**Figure 1:**
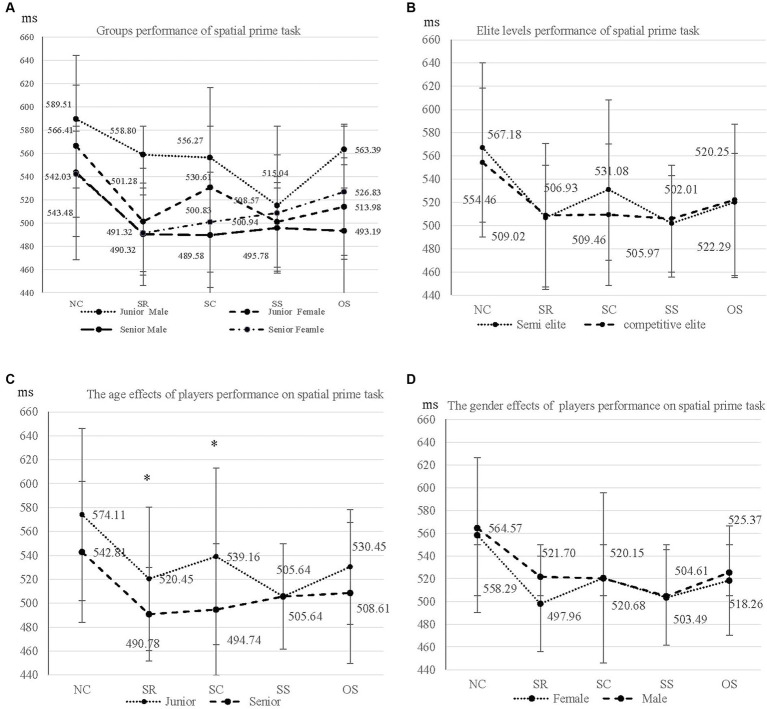
Badminton player of groups and gender differences spatial prime task performances. NC, trials was no cue; SR, cue appear in the same row; SC, cue appear in the same column; SS, cue appear in the same square; OS, cue appear in a different row. * *p* < 0.05.

**Figure 2:**
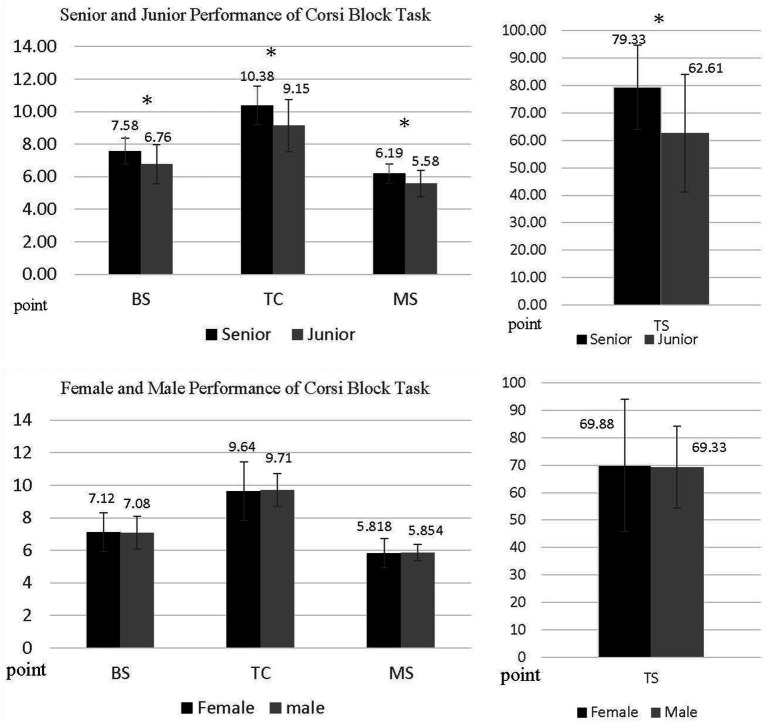
Badminton player of groups and gender differences Corsi block task performances. BS, block span; TS, total score; TC, total correct trails; MS, memory span. * *p* < 0.05.

In the SPT task, the MANOVA revealed no significant main effect for the SPT of gender, with Wilks’ lambda = 0.872, *F* (5, 49) = 1.432, *p* = 0.22, *η*^2^ = 0.128 and Observed power = 0.46; male and female players revealed the same RT. Consequently, the significant main effect of age was observed with Wilks’ lambda = 0.800, *F* (5, 49) = 2.445, *p* = *0*.047*, η*^2^ = 0.20, and Observed power = 0.72, indicating that senior high badminton players revealed faster RT than junior high players did. There was no significant interaction between gender and age group with Wilks’ lambda = 0.836, *F* (5, 49) = 1.927, *p* = *0*.107, *η*^2^ = *0*.164, and Observed power = 0.60. Group performance of SPT is shown in Table 2 and Figure 1. In the CCT task, there are no significant main effect in non-parametric MANOVA tested in gender, Wilks’ lambda = 0.977, *F* (3, 51) = 0.394, *p* = 0.758*, η*^2^ = 0.02, Observed power = 0.12, Male and Female players revealed the same span of CCT task, subsequently, age effect in Wilks’ lambda = 0.756, *F* (3, 51) = 5.489, *p* = 0.002*, η*^2^ = 0.244, Observed power = 0.92, senior high badminton player revealed longer CCT Span than junior high players, and no interaction between gender and age factors, Wilks’ lambda = 0.990, *F* (3, 51) = 0.177, *p* = 0.911*, η*^2^ = 0.010, Observed power = 0.81, respectively, groups performance of CCT are shown in Table 3 and Figure 2. Given the age effect observed in task performance, we included age as a covariance, with semi-elite and competitive-elite youth athletes as fixed variables in the SPT and CCT. The result indicated that for the SPT there was no significant difference in the performance of SPT when comparing semi-elite to competitive-elite youth athletes, with Wilks’ lambda = 0.954, *F* (5, 51) = 0.490, *p* = *0*.782, *η*^2^ = *0*.046, and Observed power = 0.15; competitive-elite and semi-elite players revealed the same RT in SPT. In the CCT, semi-elite and competitive-elite revealed a significant effect on performance, with Wilks’ lambda = 0.847, *F* (3, 53) = 3.137, *p* = *0*.03, *η*^2^ = *0*.153, Observed power = 0.69; competitive-elite players revealed longer span of CCT than semi-elite players. The elite levels of the youth athletes is illustrated in Table 1. MANCOVA was used to analyze the effects of WM in semi-elite and competitive-elite youth athletes, and the same statistical method was employed for the spatial priming task.

**Table 2:** Age and gender, perceptual and cognitive skills performance in adolescent badminton athletes.

MANOVA		Age*		Gender	
Measure		Junior = 33	Senior = 24	Female = 33	Male = 24
Spatial-Prime (ms)	NC	574.11 ± 72	542.81 ± 59	558.28 ± 68	564.57 ± 71
	SR	520.45 ± 60	490.77 ± 39	497.95 ± 42	521.70 ± 65
	SC	539.16 ± 74	494.73 ± 55	520.68 ± 75	520.14 ± 64
	SS	505.64 ± 44	501.63 ± 44	503.48 ± 42	504.60 ± 46
	OS	530.45 ± 48	508.60 ± 59	518.26 ± 48	525.36 ± 62
Corsi-Block (Score)	BS	6.76 ± 1.2	7.58 ± 0.8	7.12 ± 1.2	7.08 ± 1.01
	TS	62.61 ± 21.47	79.33 ± 15.4	69.88 ± 24.1	69.33 ± 15.0
	TC	9.15 ± 1.62	10.38 ± 1.2	9.64 ± 1.8	9.71 ± 1.04
	MS	5.57 ± 0.8	6.18 ± 0.6	5.81 ± 0.9	5.85 ± 0.5

Spatial-Prime (ms): NC, trials was no cue; SR, cue appear in the same row; SC, cue appear in the same column; SS, cue appear in the same square; OS, cue appear in a different row. Corsi-Block (Score): BS, block span; TS, total score; TC, total correct trails; MS, memory span.*p < 0.05.

**Table 3:** Sports performance, and perceptual and cognitive skills in adolescent badminton athletes.

MANCOVA		Elite level	
Measure		Semi-elite = 29	Competitive-elite = 28
Spatial-prime (ms)	NC	567.18 ± 73	554.46 ± 64
	SR	506.93 ± 45	509.01 ± 62
	SC	531.08 ± 77	509.45 ± 61
	SS	502.01 ± 41	505.96 ± 46
	OS	520.25 ± 42	522.28 ± 65

MANCOVA		Elite level *	
Corsi-Block (Score)	BS *	6.55 ± 1.21	7.68 ± 0.81
	TS	61.79 ± 22.21	77.79 ± 15.68
	TC	9.21 ± 1.69	10.14 ± 1.29
	MS	5. 60 ± 0.87	6.07 ± 0.64

Spatial-Prime (ms): NC, trials was no cue; SR, cue appear in the same row; SC, cue appear in the same column; SS, cue appear in the same square; OS, cue appear in a different row. Corsi-Block (Score): BS, block span; TS, total score; TC, total correct trails; MS, memory span.*p < 0.05.

## 4 Discussion

This study primarily aimed to examine whether there are differences in perceptual-cognitive skills in terms of WM and SPT-RTs among adolescent badminton athletes based on age and gender. We also analyzed the effect between perceptual-cognitive skills and elite levels at the semi-elite and competitive-elite levels in badminton adolescent athletes. The discussions are as follows.

### 4.1 Perceptual-cognitive skills of SPT-RT

In our results of SPT, no gender effect was observed in the groups of female and male badminton players; however, age had a significant effect on the senior and junior high groups, as senior players exhibited better performance in terms of RT than junior high players did. From the perspective of maturation (Goodway et al., 2021), the age maturation affects players’ RTs, and research has shown that age affects the timing of response initiation in sports, with older age groups exhibiting less RT than younger age groups. Söğüt and Koçak (2009) indicated that older child players (10 years old) had significantly higher scores in detection time than those of 8-year-olds. Benguigui and Ripoll (1998) show that age affects the timing of detection and has an inverse correlation. In line with previous research, our results showed that senior players, owing to their developmental maturity, exhibited better RT than junior players during adolescence. The RT decreases as age increases into adulthood (Petrakis, 1985; Williams and Ericsson, 2005). The results of the SPT of five cueing conditions revealed a similar trend to that observed by Nelson (2013). The slowest RT was observed in the NC condition (800 ms), followed by the SC condition (750 ms). The results indicated that senior high school athletes exhibited significantly faster reaction times than junior high school athletes in both the SR (490 ms) and SC (494 ms) conditions. Additionally, the CCT task resulted in a higher memory span in senior athletes than in junior athletes. The results presented here are in accordance with the findings of Furley and Memmert (2013), which indicate that WM capacity is a crucial role of an individual’s capacity to control attention and to operate efficiently in the transition of information processing (Eccles, 2006; Ericsson, 2006; Furley P. and Memmert, 2010).

In terms of gender differences, our results showed no gender effect in badminton players when performing SPT-RT, even though female athletes mature faster than their male counterparts during adolescence (Goodway et al., 2021). Additionally, with the introduction of hormones until the age of 4–9 years, boys could be more successful than girls in terms of detection time (Petrakis, 1985). This trend continues into adulthood, as males consistently outperform females (Brady, 1996; Caccese et al., 2020). However, our results revealed that both males and females performed equally well in the RT task, regardless of gender. From the perspective of training, deliberation in practice could reduce gender differences in the ability of cognitive (Chance and Goldstein, 1971; Connor et al., 1977), specifically the detection time decreases and have better ability to select RT through badminton training (Liu et al., 2017). Furthermore, experienced athletes have better perceived time than novice athletes (Phomsoupha and Laffaye, 2015).

### 4.2 Perceptual-cognitive skills of WM Corsi-block span

In the CCT result, no gender effect was observed among the groups of female and male adolescent badminton players; however, significant differences were found based on age between the junior and senior high groups, indicating that age affected adolescent badminton players, and senior players performed better than junior badminton players, this finding is consistent with the study by Ward and Williams (2003) in which age was a stronger predictor of structured memory recall than ability. Regarding the development and gender-based research in WM, our findings correspond with the research of Farrell Pagulayan et al. (2006) result, as their study also found no gender effect but it did identify significant differences based on grade. Consistent with the results of Rigoli et al. (2012), research investigating motor coordination, academic achievement, and WM also showed no gender effects in adolescent participants. Elite players blend contextual information with stored memory in a way that differs systematically from their sub-elite peers (Ward and Williams, 2003).

### 4.3 Elite levels level and perceptual-cognitive level

**Figure 3:**
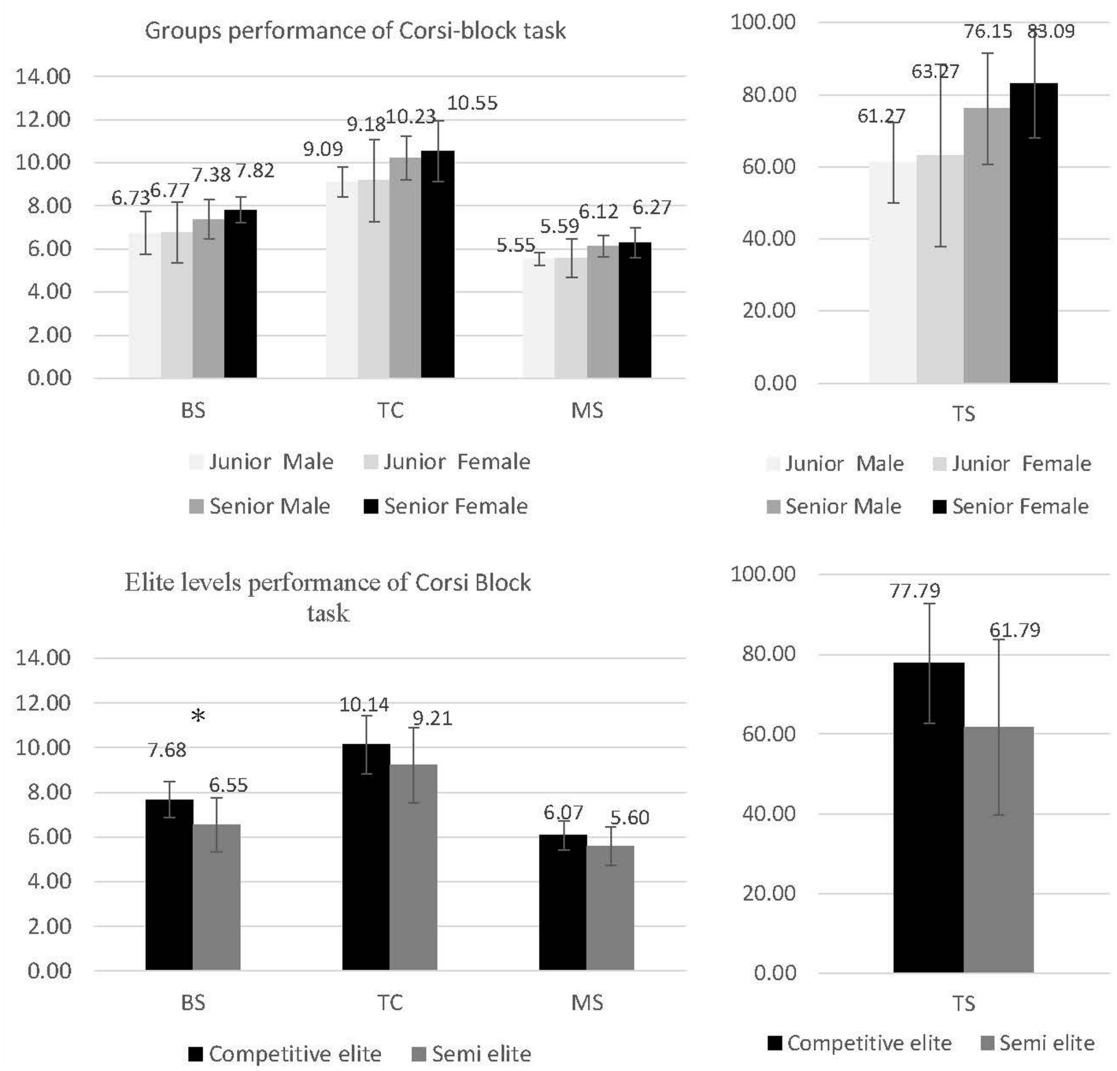
Badminton player of elite levels Corsi block task performances. Semi elites *n* = 29, competitive elites *n* = 28 (including 2 successful elites). We combine the competitive elite athletes and successful elites. * *p* < 0.05.

In this study, we assessed the elite levels of adolescent badminton players using Swann et al. (2015) equation to classify athletes into semi-elite and competitive-elite players and analyzed the association between elite levels and perceptual-cognitive skills. The results revealed that in the SPT task, there was no difference in RT performance, which means that the selection RT was the same between semi-elite and competitive-elite players; however, regarding the WM in the CCT, competitive-elite players showed better results than semi-elite players (see Table 3; Figure 3). These findings are consistent with the Alves et al. (2013) studies and the same with athletes with higher skills performed better cognitively than athletes with lower skills (Ward and Williams, 2003; Tomporowski and Pesce, 2019), Elite athletes possess superior cognitive skills and support the concept of WM as executive attention (Engle, 2002), the viewpoint suggesting that WM capacity is linked to controlled attention and implying that WM capacity does not directly represent a person’s ability to keep items in their short-term memory. Instead, it symbolizes a person’s capacity to persist with task objectives, minimize disturbances, and avoid distractions (Engle, 2002). Furley and Memmert (2012) indicated that individuals with higher WM capabilities were notably better at maintaining focus and resisting interruptions during sports, underscoring the critical importance of WM in managing attention during athletic competitions. Additionally, Furley and Memmert (2013) illustrated how athletes’ WM content is instrumental in shifting their attentional focus, enabling them to consciously direct their attention toward achieving targeted behaviors. Further evidence points to a two-way interaction between the contents of attention and WM; attention is a gateway for stimuli to enter WM (Atkinson and Shiffrin, 1968), while researchers have found that the contents of WM can also shape attentional control (Soto and Humphreys, 2007; Soto and Humphreys, 2008). Thus, competitive athletes seem well in attention to perform well in open-skill badminton sports. From childhood through adolescence to adulthood, the greater the WM, the greater the motor processing in the development effect (Rigoli et al., 2012; Alves et al., 2013; Lehmann et al., 2014), which further indicates that higher elite levels of competitive-elite badminton players’ WM is better than lower elite levels of semi-elite adolescent players. WM capacity difference affects competition responses; higher WM capacity has more proficiency in adjusting tactical decisions, while lower WM capacity may fails to adjust tactical decisions to the demands of the game.

## 5 Limitations

The scope of this study was limited to adolescent badminton athletes; therefore, the results cannot be generalized to other sports or age groups. Additionally, the study only examined cognitive abilities related to WM and SPT-RTs. The cognitive tasks used in this study did not cover all the cognitive parameters, such as cognitive flexibility and inhibition ability, which are typically included in cognitive studies. Hence, it is crucial for future studies to incorporate more comprehensive cognitive tests to assess different aspects of cognition. Moreover, the study did not gather data on the transition from adolescence to college age, potentially affecting the outcomes of the variables.

## 6 Conclusion

In the current study, we examined the perceptual-cognitive skills of SPT-RTs and CCT-WM across gender, age and elite levels among badminton adolescent athletes. The results indicated that age affects the perceptual-cognitive skills of adolescent players. Senior high school players exhibited better perceptual-cognitive abilities than junior high school players did. No gender effects were found in the perceptual skills of the adolescents, and the age effect was consistent across genders. Regarding the different levels of elite in adolescent badminton players, SPT-RTs were similar in the semi-elite and competitive players; however, regarding the WM in CCT, competitive elite players outperformed semi-elite players. Our results show that the WM plays a crucial role in open skills in elite levels specific to badminton. There were no gender effects; only the age difference effect among adolescent badminton players was observed. Female and male adolescents of the same age perform equally well in terms of perceptual-cognitive skills in the same age. When developing advanced skills for elite levels, perceptual-cognitive skills should be considered during routine training. Future studies should focus on the potential of perceptual-cognitive expertise in sports to assist trainers in developing expert athletes and gain insight into the brain structure and functional maturation that differs between individuals with varying levels of sports experience and expertise, ultimately enhancing higher levels.

## Data availability statement

The original contributions presented in the study are included in the article/supplementary material, further inquiries can be directed to the corresponding author.

## Ethics statement

This study was conducted in accordance with the Declaration of Helsinki and approved by the Institutional Review Board of the University of Taipei (protocol code: UT-IRB No. IRB-2020-073, February 9, 2021–2024) and received written informed consent from the participants’ legal guardians. The studies were conducted in accordance with the local legislation and institutional requirements. Written informed consent for participation in this study was provided by the participants’ legal guardians/next of kin. Written informed consent was obtained from the minor(s)' legal guardian/next of kin for the publication of any potentially identifiable images or data included in this article.

## Author contributions

K-CW: Writing – original draft, Writing – review & editing. Y-LL: Data curation, Writing – original draft. S-CC: Writing – review & editing.
